# M2R: a Python add-on to cobrapy for modifying human genome-scale metabolic reconstruction using the gut microbiota models

**DOI:** 10.1093/bioinformatics/btab060

**Published:** 2021-02-01

**Authors:** Ewelina Weglarz-Tomczak, Jakub M. Tomczak, Stanley Brul

**Affiliations:** Swammerdam Institute for Life Sciences, Faculty of Science, University of Amsterdam, Amsterdam, The Netherlands; Department of Computer Science, Faculty of Science, Vrije Universiteit Amsterdam, Amsterdam, The Netherlands; Swammerdam Institute for Life Sciences, Faculty of Science, University of Amsterdam, Amsterdam, The Netherlands

## Abstract

**Motivation:**

The gut microbiota is the human body’s largest population of microorganisms that interact with human intestinal cells. They use ingested nutrients for fundamental biological processes and have important impacts on human physiology, immunity and metabolome in the gastrointestinal tract.

**Results:**

Here, we present M2R, a Python add-on to cobrapy that allows incorporating information about the gut microbiota metabolism models to human genome-scale metabolic models (GEMs) like RECON3D. The idea behind the software is to modify the lower bounds of the exchange reactions in the model using aggregated in- and out-fluxes from selected microbes. M2R enables users to quickly and easily modify the pool of the metabolites that enter and leave the GEM, which is particularly important for those looking into an analysis of the metabolic interaction between the gut microbiota and human cells and its dysregulation.

**Availability and implementation:**

M2R is freely available under an MIT License at https://github.com/e-weglarz-tomczak/m2r.

**Supplementary information:**

Supplementary data are available at *Bioinformatics* online.

## 1 Introduction

The human cell metabolism is regulated by cell-intrinsic and cell-extrinsic factors. For instance, cell-intrinsic factors are genes and hormones, while cell-extrinsic factors are associated with the environment, and include nutrients availability, tissue-specific context and microbiota that resides on or within human tissues. Genome-wide modeling provides a useful approach to uncover the molecular basis of metabolism (Mendoza *et al.*, 2019). Most comprehensive human genome-wide model (GEM) Recon3D constitutes a computational resource that includes three-dimensional (3D) metabolite and protein structure data, and enable accurate integrated analyses of metabolic functions in humans (Brunk *et al.*, 2018). Human GEMs have been extensively used for analyzing mRNA expression data to elucidate how changes in gene expression impact cell physiology (Pandey *et al.*, 2019). However, genetics is not the only determinant of nutrient utilization by human cells. Metabolism is also influenced by metabolic constraints imposed by the environmental context. Integration of the nutrients availability data into genome-wide models improves prediction of metabolic phenotypes (Węglarz-Tomczak *et al.*, 2020; Zampieri *et al.*, 2019). From the human metabolism perspective, the gut microbiota is especially important (Gentile and Weir, 2018). Its role is apparent in the proper functioning of the host body and any abnormalities of their constitution could lead to diseases like colon cancer (Wong and Yu, 2019). All these facts clearly show that modeling of the human metabolism in isolation from external factors like the gut microbiota is at best only partially true. Therefore, there is an increasing interest in combining human and microbe models (Baldini *et al.*, 2019; Thiele *et al.*, 2020).

Here, we consider a problem of the host-microbiota co-metabolism and the influence of the gut microbiota on the pool of metabolites available to a host. The goal of this work is to provide a method and software that modifies a human genome-scale metabolic model (e.g. Recon3D) using information about in- and out-fluxes obtained through the Flux Balance Analysis (Orth *et al.*, 2010) from the chosen gut microbiota metabolic model(s). The software is a stand-alone Python add-on to cobrapy (Ebrahim *et al.*, 2013), a standard framework for constraint-based modeling of metabolism. It loads metabolic models of the gut microbe(s) provided by a user and adapts lower bounds of the exchange reactions in a genome-wide reconstruction model that contains metabolites associated with the gut microbe(s). We refer to this software as Microbiota-to-Recon (M2R).

## 2 Methods and features

M2R consists of five steps: (i) loading microbiota models, (ii) reading in- and out-fluxes of metabolites, (iii) aggregating all in- and out-fluxes, (iv) normalizing all in- and out-fluxes and (v) modifying reactions in a genome-scale reconstruction metabolic model that contain metabolites present in the loaded gut microbiota models. A schematic representation of our framework is presented inFigure 1. M2R is implemented in Python (the code could be run using Python 2.x or Python 3.x), using cobrapy for loading, writing and processing models.

**Fig. 1.**
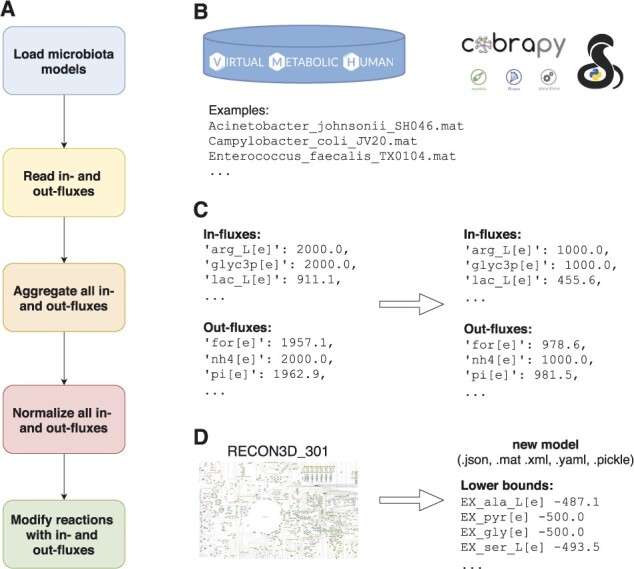
A schematic representation of our framework. (**A**) Five steps in M2R: (i) loading microbiota models from a folder specified by a user, (ii) reading in- and out-fluxes of all metabolites from microbe(s) models, (iii) aggregating in- and out-fluxes of all metabolites, (iv) normalizing values of in- and out-fluxes of metabolites to a value specified by a user, (v) modifying lower bounds of reactions in a model with normalized in- and out-fluxes. (**B**) Software components used in our program: Virtual Metabolic Human database for microbiota models, and cobrapy for processing models. (**C**) Examples of results for normalizing fluxes. (**D**) Examples of results of modified reaction lower bounds in a model

The main idea of the presented program is to modify a metabolic model by taking into account information of metabolites that take part in the external reactions of the selected gut microbiota models. Typically, genome-scale metabolic models are assumed to be in their steady-state. Hence, it is possible to consider only those metabolites of the gut microbe(s) that are part of their external reactions, because these are responsible for the consumption or production of nutrients.

In analyses based on fluxes, a lower/upper bound of a reaction determines a possible uptake/secretion. Further, if there is no prior knowledge available like gene expressions or proteomics, and/or nutrients availability data the lower/upper bounds of reactions in the model are typically set to an arbitrary value, e.g. 1000.

The gut microbiota could consume nutrients or produce metabolites, thus, we can use this information to modify the lower bounds of the reactions in the host model. First, we need to read and aggregate fluxes of the microbe(s) metabolites associated with the microbe(s) external reactions. Then, we normalize them to a given value, i.e. by dividing aggregated in- and out-fluxes by the maximum value of all fluxes and then multiplying them by the desired value, e.g. 1000. Finally, we update the lower bounds associated with the microbe(s) metabolites as follows:

(1) $$LB_{r}:=\alpha LB_{r}+(1-\alpha )f_{i}-(1-\alpha )f_{o},$$

where *LB_r_* is a lower-bound of a reaction *r*, *f_i_* and *f_o_* denote an in-flux and an out-flux of the considered gut microbe(s) metabolite involved in the reaction *r*, respectively, and α∈[0,1] is a weight that scales the original value of the lower bound and the in- and out-fluxes. Note that in most cases *f_i_* and/or *f_o_* equals 0.

We introduce the factor *α* that defines the weight of the biological systems. Since it is estimated that in a human body there are at least as many microbes as nucleated human cells, we set *α* to 0.5 in our demonstration.

## 3 Conclusion

In this work, we present Python-based software for modifying the human genome-scale metabolic model with the gut microbe(s) model(s). Its development in Python, an open-source platform, simplifies distribution and enables the use on most operating systems. M2R is easy-to-use and allows us to update the pool of nutrients/metabolites available for the host based on the gut microbiota metabolism. Lastly, the code is freely available and it could be further developed in the future.

## Funding

E.W.-T. was financed by a grant within Mobilność Plus V from the Polish Ministry of Science and Higher Education [1639/MOB/V/2017/0].

*Conflict of Interest*: none declared.

## Supplementary Material

btab060_Supplementary_Data
