# Supplementary material for: M2R: a Python add-on to cobrapy for modifying human genome-scale metabolic reconstruction using the gut microbiota models
Source: Bioinformatics. 2021 Feb 1;37(17):2785–6. doi: 10.1093/bioinformatics/btab060 (PMC8428599; doi:10.1093/bioinformatics/btab060)
Supplement: btab060_Supplementary_Data [file btab060_supplementary_data.pdf]

# SUPPLEMENTARY DATA
for
# M2R: a Python add-on to cobrapy for modifying human genome-scale metabolic reconstruction using the gut microbiota models

Ewelina Weglarz-Tomczak[1,*], Jakub M. Tomczak[2,*] and Stanley Brul[1]

[1]Swammerdam Institute for Life Sciences, Faculty of Science, University of Amsterdam, the Netherlands
[2]Department of Computer Science, Faculty of Science, Vrije Universiteit Amsterdam, the Netherlands
Contact: `ewelina.weglarz.tomczak@gmail.com`

## Contents

**1 An example of use: *Trueperella pyogenes MS249* and *Vibrio fluvialis 560*** **2**
1.1 Glycolysis pathway . . . . . 3
1.2 Exchange reactions . . . . . 5
1.3 Biomass . . . . . 7

**2 A demonstration** **9**
2.1 Introduction . . . . . 9
2.2 Running M2R . . . . . 9
2.2.1 Providing information to the program . . . . . 9
2.2.2 Aggregating in- and out-fluxes and normalization . . . . . 10
2.2.3 Modifying lower bounds of the model . . . . . 11
2.3 Remarks . . . . . 11

# 1 An example of use: *Trueperella pyogenes MS249* and *Vibrio fluvialis 560*

In order to provide an insight into the proposed method, we present a flux-based analysis of the impact of *Trueperella pyogenes MS249* and *Vibrio fluvialis 560* on the human genome-scale metabolic reconstruction (`Recon3DModel_301`). In this experiment, these two gut microbiota were selected at random. Our goal is to show differences in fluxes distribution in a host model modified by `M2R`.

In the presented analysis, we used `biomass_maintenance` as the objective function for the Flux Balance Analysis (FBA). We consider four models with $\alpha = 0.5$:

1. `Recon`: The result of `M2R` if no microbiota is provided.
2. `Recon+T`: The result of `M2R` with *Trueperella pyogenes MS249*.
3. `Recon+V`: The result of `M2R` with *Vibrio fluvialis 560*.
4. `Recon+TV`: The result of `M2R` with *Trueperella pyogenes MS249* and *Vibrio fluvialis 560*.

We present flux distributions for:

- Glycolysis pathway (Subsection 1.1);
- Selected exchange reactions (Subsection 1.2);
- Biomass objective function (Subsection 1.3).

We used `Escher`[1] for visualizing the fluxes, together with `CobraPy`[2] for FBA.

[1] https://escher.readthedocs.io/
[2] https://cobrapy.readthedocs.io/

## 1.1 Glycolysis pathway

In Figures 1, 2, 3 and 4 the flux distribution of glycolysis pathway for, respectively, `Recon`, `Recon+T`, `Recon+V` and `Recon+TV` are presented. A closer inspection of the distributions indicates that indeed including selected gut microbiota results in different fluxes. For instance, including *Trueperella pyogenes MS249* alone causes the flux of *LDH_L* increases from −500 in `Recon` to 0 in `Recon+T`. Similarly, the flux of *ASPTAm* drops from 150 to 0. Interestingly, inclusion of both *Trueperella pyogenes MS249* and *Vibrio fluvialis 560* results in a flux distribution that partially resemblences the flux distribution of `Recon+T` and partially matches the flux distribution of `Recon+V`.

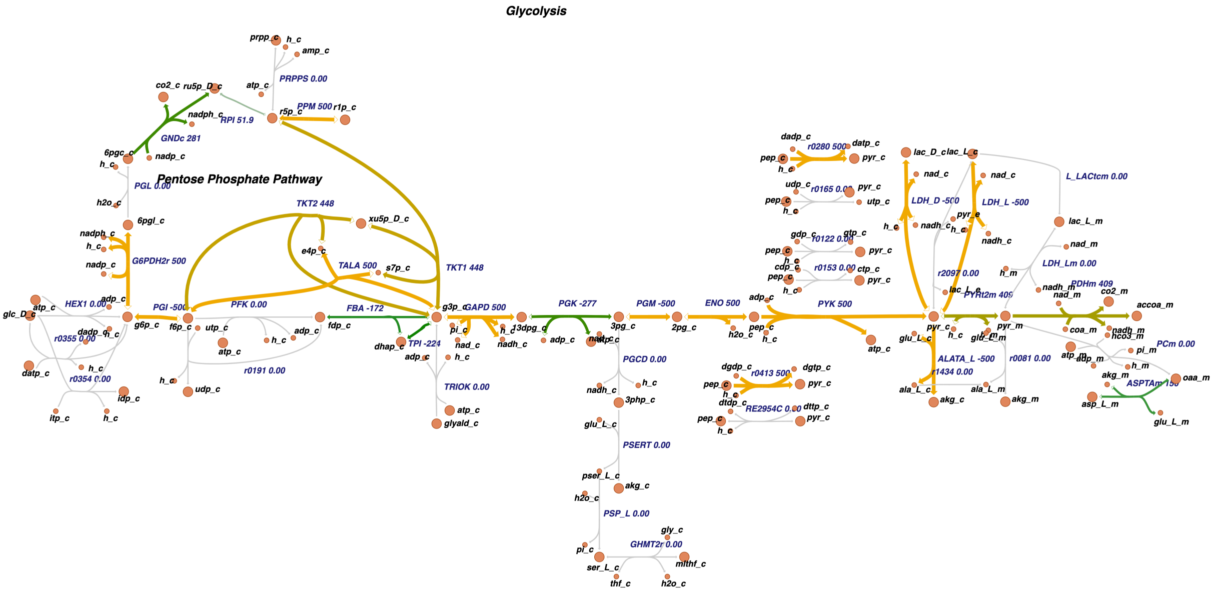


Figure 1: The flux distribution of glycolysis pathway for `Recon`

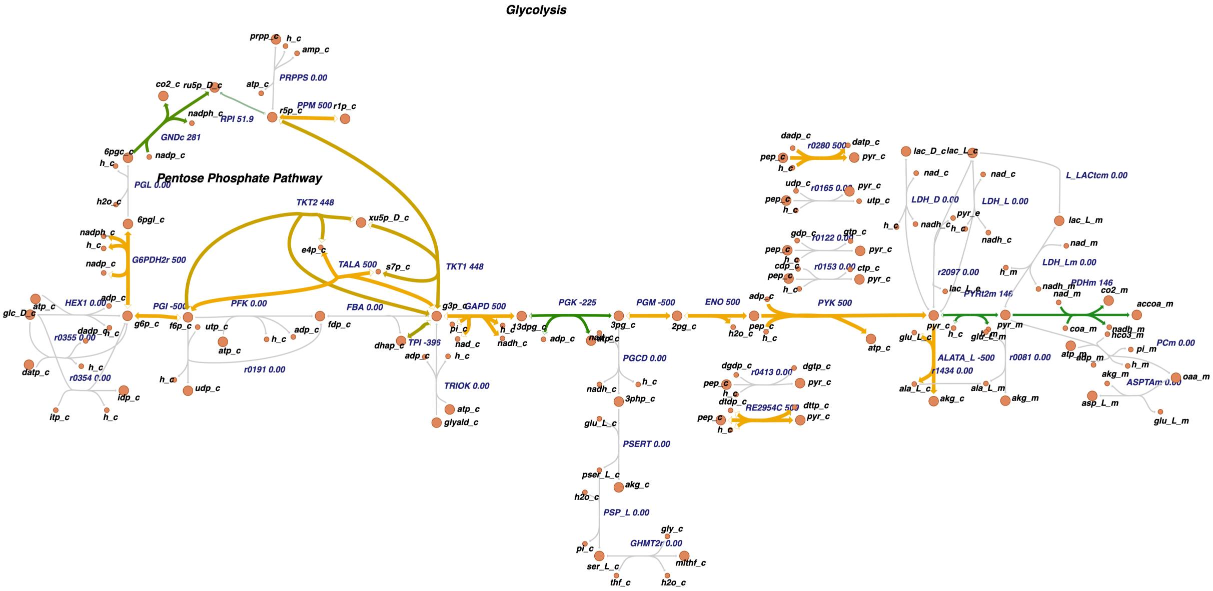


Figure 2: The flux distribution of glycolysis pathway for `Recon+T`

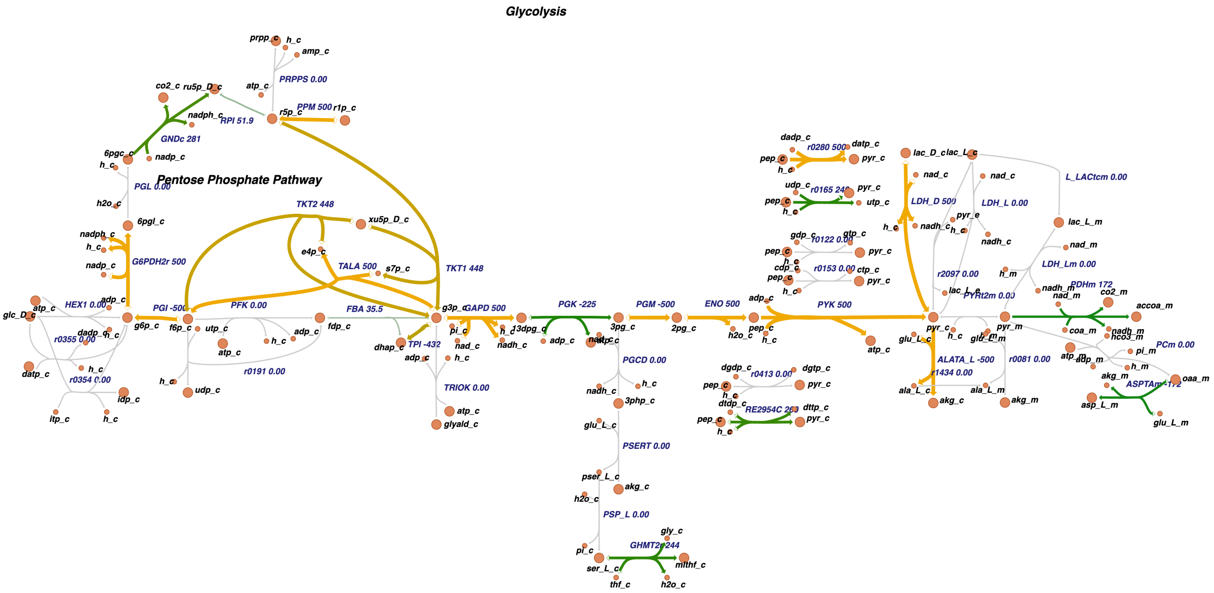


Figure 3: The flux distribution of glycolysis pathway for `Recon+V`

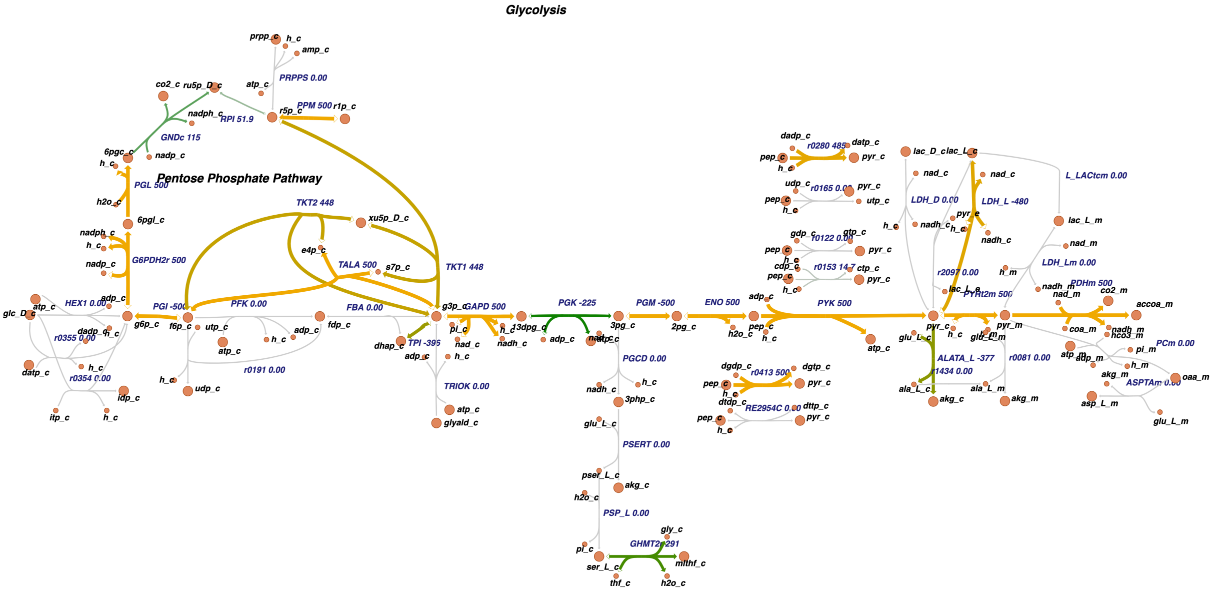


Figure 4: The flux distribution of glycolysis pathway for `Recon+TV`

## 1.2 Exchange reactions

In Figures 5, 6, 7 and 8 the flux distribution of exchange reactions for, respectively, `Recon`, `Recon+T`, `Recon+V` and `Recon+TV` are presented. Here, the differences among flux distributions are more apparent than in the case of glycolysis pathway. For instance, $EX\_glc\_D\_e$ varies from 305 for `Recon` to 0 (`Recon+T`), 6.6 (`Recon+V`) and 0 (`Recon+TV`). Another significant difference is for $EX\_o2\_e$ that increases from $-500$ for `Recon` to $-314$ (`Recon+T`), $-101$ (`Recon+V`) and $-208$ (`Recon+TV`). There are more differences, however, our goal is only to visualize that there are differences rather than providing in-depth analysis.

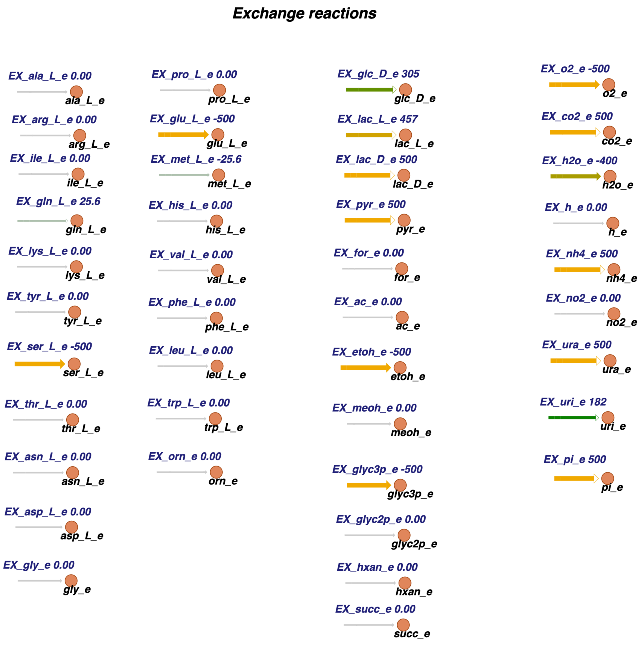


Figure 5: The flux distribution of exchange reactions for `Recon`

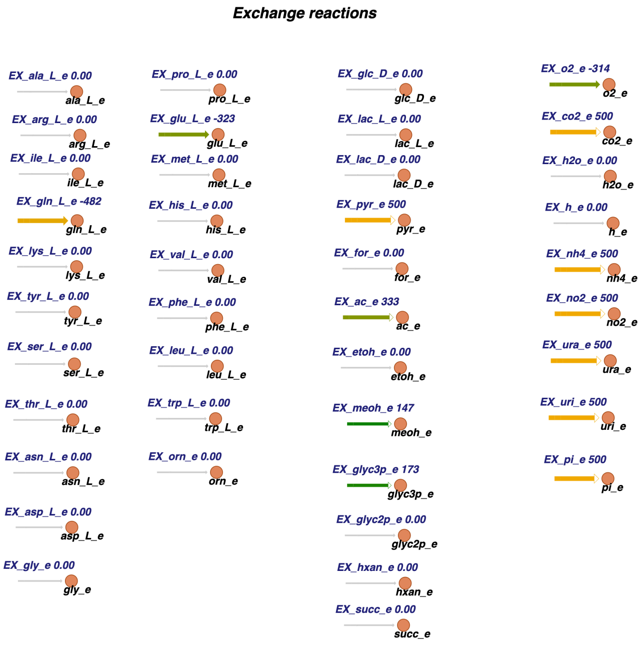


Figure 6: The flux distribution of exchange reactions for `Recon+T`

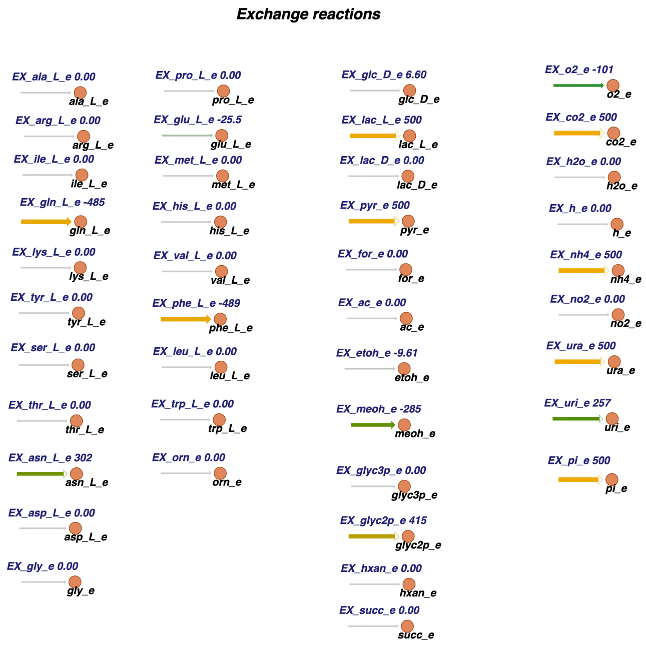


Figure 7: The flux distribution of exchange reactions for `Recon+V`

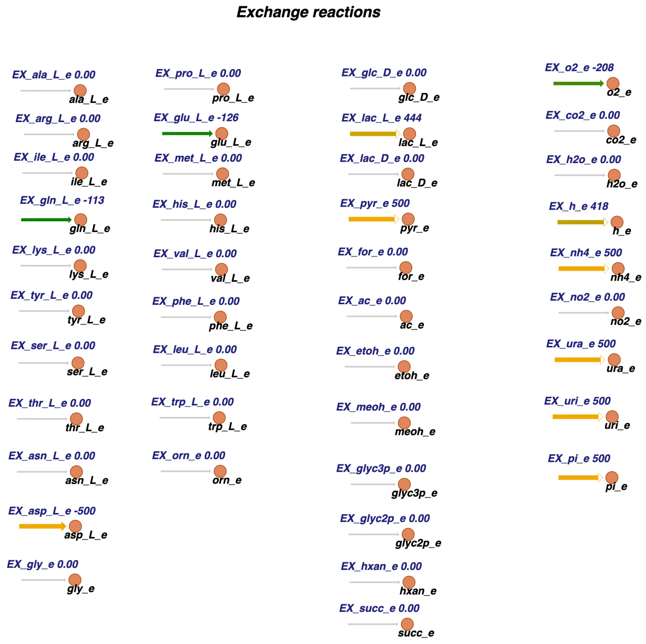


Figure 8: The flux distribution of exchange reactions for `Recon+TV`

## 1.3 Biomass

In Figures 9, 10, 11 and 12 the flux distribution of the biomass reaction for, respectively, `Recon`, `Recon+T`, `Recon+V` and `Recon+TV` are presented. In all cases, the biomass reaction value equals 378.

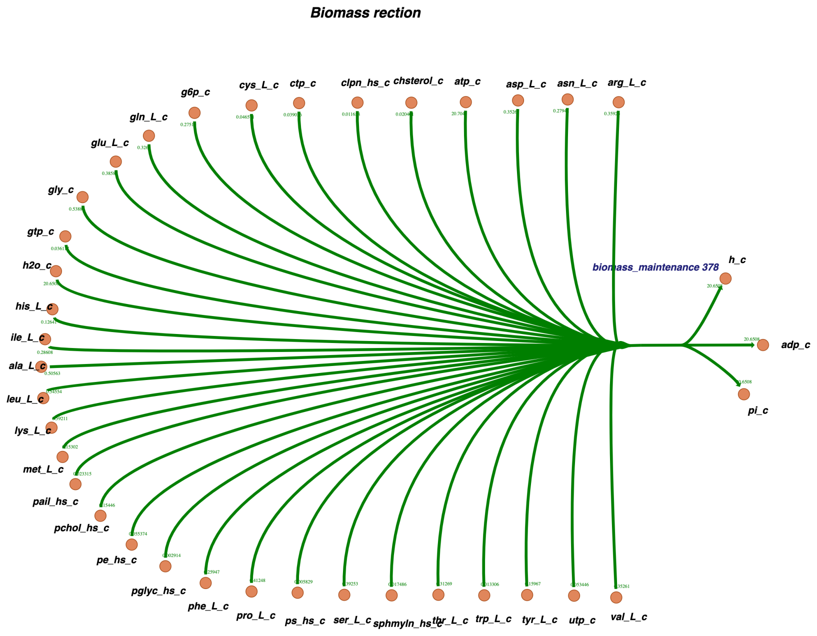


Figure 9: The flux distribution of the biomass reaction for `Recon`

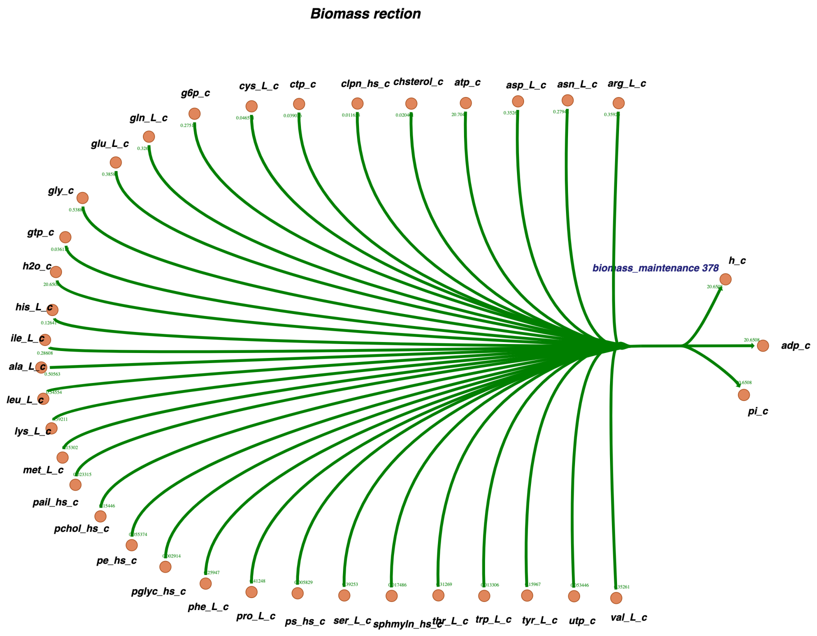


Figure 10: The flux distribution of the biomass reaction for `Recon+T`

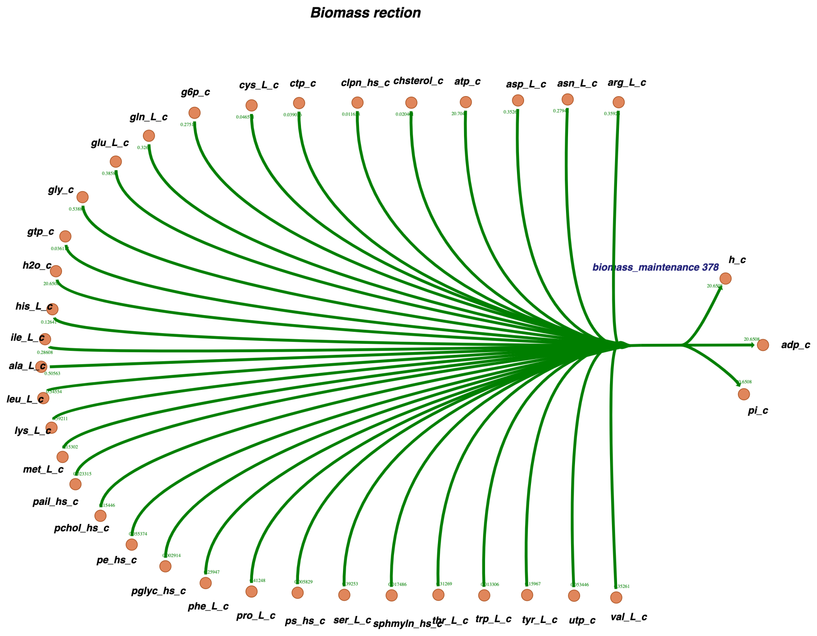


Figure 11: The flux distribution of the biomass reaction for `Recon+V`

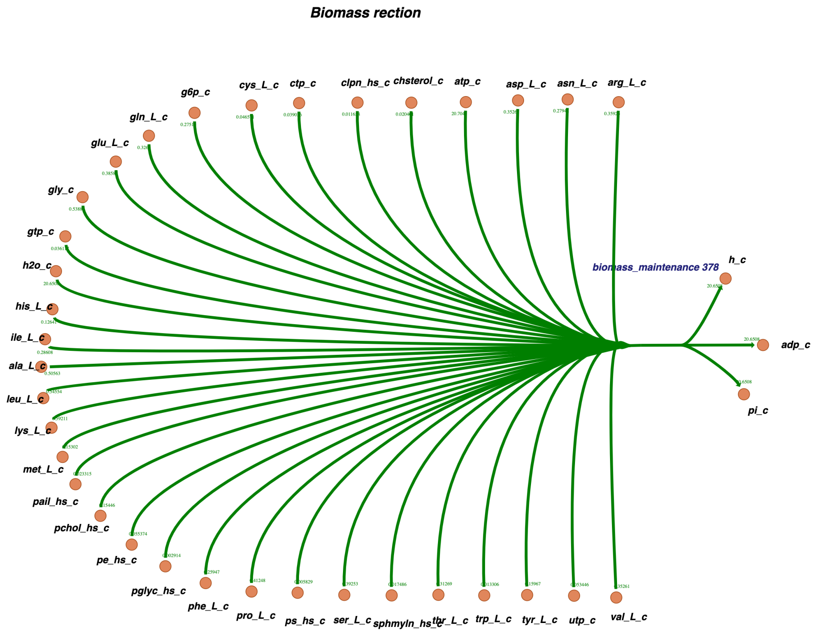


Figure 12: The flux distribution of the biomass reaction for `Recon+TV`

# 2 A demonstration

## 2.1 Introduction

The M2R program is freely available online at: `https://github.com/e-weglarz-tomczak/m2r`. It is a self-contained add-on, namely, it could be run in a console by typing:
`python m2r.py`
or
`python3 m2r.py`
Alternatively, it could be run in a Python IDE.

Additionally, we provide an example of using `M2R` in a `jupyter` notebook file `m2r_example.ipynb` where all separate steps are given. In this document, we show results for all steps.

In this demonstration, we use `Recon3D_301.pickle` that is created from `Recon3D_301.mat` obtained from `https://www.vmh.life/`, and two randomly selected gut microbiota models (`Trueperella_pyogenes_MS249.pickle`, `Vibrio_fluvialis_560.pickle`) also obtained from `https://www.vmh.life/`.

## 2.2 Running M2R

### 2.2.1 Providing information to the program

After starting the program, a welcome message appears followed by messages about providing information about a directory with gut microbiota models, a directory with a human genome-scale metabolic model, a normalization value, an $\alpha$ value, and a name of a new metabolic model.

```
Welcome to Microbiota-to-Recon (M2R) program!

 ***************************
 * __   __   ___     ____  *
 *|  \ /  | / _ \   |  _ \ *
 *|   ^   | \/ \ |  | |_|/ *
 *|  | |  |   / /_  |   \  *
 *|__| |__|  /____\ |_|\_\ *
 *                         *
 ***************************
© E. Weglarz-Tomczak & J. Tomczak

This program consists of three steps that modify RECON
by introducing information from microbiota.

------------------

Please provide a folder name where the microbiota files (.mat or .pickle) are (default: microbiota, type 0 if you wan
t to skip loading microbiota):
         -> microbiota
Please provide a list of names of microbiota that are supposed to influence the model separated by a space (e.g., Rum
inococcus_sp_5_1_39BFAA.mat Acinetobacter_baumannii_AB0057.mat, default: load all files from the folder):
         -> all files in the directory will be loaded
Please provide a value of alpha (between 0 and 1; default: 0.5): 0.5
         -> 0.5
Please provide a value that fluxes should be normalized to (default: 1000.): 1000.
         -> 1000.0
Please provide a directory name where the model is (default: models):
         -> models
Please provide a name of the model (please rememeber about the extension: either .mat, .json, .xml, .yaml or .pickl
e): Recon3DModel_301.pickle
         -> Recon3DModel_301.pickle
         Loading the model (this may take a while)...
                 ...success!
Please provide a name for the new model (possible extensions: .mat, .json, .xml, .yaml or .pickle, e.g., New_recon.ma
t): new.json
         -> new.json

All is set up!
Let`s start!

------------------
```

### 2.2.2 Aggregating in- and out-fluxes and normalization

The next step is the aggregation of the gut microbiota metabolite in- and out-fluxes:

```
ins, outs = m2r.calculate_ins_outs(m2r.files, m2r.folder)
```

```
Step 1/3: Read microbiota...
        (File 1 out of 2) Vibrio_fluvialis_560.pickle
        (File 2 out of 2) Trueperella_pyogenes_MS249.pickle
        ...done!
```

The function above returns the following:

```
ins
```

```
{'arg_L[e]': 2000.0,
 'glyc3p[e]': 2000.0,
 'gcald[e]': 998.7315731829896,
 '3mop[e]': 865.2223285129872,
 'o2[e]': 1169.7545965403626,
 'uri[e]': 409.4140620067445,
 'cit[e]': 329.885644654054,
 'thr_L[e]': 261.9377310426362,
 'no2[e]': 79.41516839853418,
 'lac_L[e]': 72.52867426598105,
 'man[e]': 64.79422393696815,
 'amp[e]': 147.84261295087944,
 'val_L[e]': 48.094943512086246,
 'glyleu[e]': 46.18093598666063,
 'lys_L[e]': 38.97516019671778,
 'gln_L[e]': 66.84122206004419,
 'cys_L[e]': 29.11605861871921,
 'asn_L[e]': 50.43612552437274,
 'pro_L[e]': 46.29736463883938,
 'dgsn[e]': 22.7952936920577,
 'phe_L[e]': 21.098800986535974,
 'ttdca[e]': 47.448933081260236,
 'sucr[e]': 17.075527811100553,
 'tyr_L[e]': 564.1640809200885,
 'etha[e]': 12.220905620607915,
 'ocdca[e]': 11.631376282921826,
 'cytd[e]': 23.318860017281583,
 'his_L[e]': 10.823034955971965,
 'h2o[e]': 995.3945651221853,
 '2obut[e]': 753.1042240074249,
 'hxan[e]': 309.8952654430645,
 'gly[e]': 55.5345557753963,
 'ala_L[e]': 51.90443880133416,
 'leu_L[e]': 43.01753932991899,
 '26dap_M[e]': 35.22106154127062,
 'ins[e]': 30.57027641061106,
 'ser_L[e]': 27.474145770151218,
 'glu_L[e]': 16.508105339114422,
 'met_L[e]': 15.354828704637399}
```

```
outs
```

```
{'ac[e]': 1155.3090638039348,
 'ala_L[e]': 1000.0,
 'co2[e]': 1618.9490751214482,
 'h[e]': 1514.5932673378657,
 'ppa[e]': 1705.8721369765128,
 'pi[e]': 1943.8995416520306,
 'ser_L[e]': 941.2500390999842,
 'orn[e]': 1776.6775983438074,
 'dcyt[e]': 380.7533925176661,
 'for[e]': 1194.862078297211,
 'mal_L[e]': 166.30207456929648,
 'akg[e]': 121.76182943559508,
 'pyr[e]': 114.96222451827033,
 'dad_2[e]': 35.01619931266562,
 'nh4[e]': 1000.0,
 'phe_L[e]': 516.1495933326695,
 'asp_L[e]': 510.1330508903062,
 'gua[e]': 324.9483330823595,
 'ade[e]': 76.82760174556631}
```

After the aggregation, we run the normalization step:

```
ins_n, outs_n = m2r.normalize_ins_outs(ins, outs)
```

```
Step 2/3:  Normalize metabolites...
        ... done!
```

that returns the following:

```
ins_n
```

```
{'arg_L[e]': 1000.0,
 'glyc3p[e]': 1000.0,
 'gcald[e]': 499.3657865914948,
 '3mop[e]': 432.6111642564936,
 'o2[e]': 584.8772982701813,
 'uri[e]': 204.70703100337224,
 'cit[e]': 164.942822327027,
 'thr_L[e]': 130.9688655213181,
 'no2[e]': 39.70758419926709,
 'lac_L[e]': 36.264337132990526,
 'man[e]': 32.397111968484076,
 'amp[e]': 73.92130647543972,
 'val_L[e]': 24.047471756043123,
 'glyleu[e]': 23.090467993330314,
 'lys_L[e]': 19.48758009835889,
 'gln_L[e]': 33.420611030022094,
 'cys_L[e]': 14.558029309359606,
 'asn_L[e]': 25.21806276218637,
 'pro_L[e]': 23.14868231941969,
 'dgsn[e]': 11.39764684602885,
 'phe_L[e]': 10.549400493267987,
 'ttdca[e]': 23.724466540630118,
 'sucr[e]': 8.537763905550277,
 'tyr_L[e]': 282.08204046004425,
 'etha[e]': 6.110452810303958,
 'ocdca[e]': 5.815688141460913,
 'cytd[e]': 11.659430008640792,
 'his_L[e]': 5.411517477985982,
 'h2o[e]': 497.69728256109266,
 '2obut[e]': 376.55211200371247,
 'hxan[e]': 154.94763272153224,
 'gly[e]': 27.76727788769815,
 'ala_L[e]': 25.95221940066708,
 'leu_L[e]': 21.508769664959495,
 '26dap_M[e]': 17.61053077063531,
 'ins[e]': 15.28513820530553,
 'ser_L[e]': 13.737072885075609,
 'glu_L[e]': 8.254052669557211,
 'met_L[e]': 7.677414352318699}
```

```
outs_n
```

```
{'ac[e]': 577.6545319019674,
 'ala_L[e]': 500.0,
 'co2[e]': 809.4745375607241,
 'h[e]': 757.2966336689328,
 'ppa[e]': 852.9360684882564,
 'pi[e]': 971.9497708260153,
 'ser_L[e]': 470.6250195499921,
 'orn[e]': 888.3387991719037,
 'dcyt[e]': 190.37669625883305,
 'for[e]': 597.4310391486055,
 'mal_L[e]': 83.15103728464824,
 'akg[e]': 60.88091471779754,
 'pyr[e]': 57.481112259135166,
 'dad_2[e]': 17.50809965633281,
 'nh4[e]': 500.0,
 'phe_L[e]': 258.07479666633475,
 'asp_L[e]': 255.06652544515313,
 'gua[e]': 162.47416654117976,
 'ade[e]': 38.413800872783156}
```

### 2.2.3 Modifying lower bounds of the model

The last step is about modifying the lower bounds of the metabolic model:

```
Recon = m2r.modify_recon(m2r.R, ins_n, outs_n, alpha=m2r.alpha)
```

```
Step 3/3:  Modify the model...
         ... done!
```

An exemplary reaction with modified lower bound value is presented below (left: before the change, right: after the change):

```
m2r.R.reactions.get_by_id('EX_glu_L[e]')
```

| | |
|---|---|
| **Reaction identifier** | EX_glu_L[e] |
| **Name** | Exchange of L-Glutamate |
| **Memory address** | 0x07faa5fa9f210 |
| **Stoichiometry** | glu_L[e] <=><br>L-Glutamate <=> |
| **GPR** | |
| **Lower bound** | -1000.0 |
| **Upper bound** | 1000.0 |

```
Recon.reactions.get_by_id('EX_glu_L[e]')
```

| | |
|---|---|
| **Reaction identifier** | EX_glu_L[e] |
| **Name** | Exchange of L-Glutamate |
| **Memory address** | 0x07faa6571f450 |
| **Stoichiometry** | glu_L[e] <=><br>L-Glutamate <=> |
| **GPR** | |
| **Lower bound** | -495.87297366522137 |
| **Upper bound** | 500.0 |

## 2.3 Remarks

- The jupyter notebook indicates partial results so that a user can understand each step.
- The program is self-contained and outputs a modified model.
- The proposed approach is implemented as a Python class that could be extended or modified.
- The code is provided under the MIT License.
